# Access to Healthcare Facilities and Women’s Healthcare Requirements in Urban Areas: A Case Study of Beijing

**DOI:** 10.3390/ijerph19063709

**Published:** 2022-03-21

**Authors:** Yingyi Zhang

**Affiliations:** School of Architecture and Urban Planning, Beijing University of Civil Engineering and Architecture, Beijing 100044, China; zhangyingyi@bucea.edu.cn; Tel.: +86-159-2179-3823

**Keywords:** healthcare facilities, accessibility, women requirements, Healthy City, gender equality

## Abstract

Access to healthcare facilities is an essential measure of the urban development of contemporary cities. Governments often budget huge sums to fulfill the healthcare demands of the population, however neglect to address requirements specific to women. This paper assesses the density, spatial distribution, and services of healthcare facilities to identify care requirements specific to women, and how their needs are—or could be—met. The analysis addresses the research question: What strategies will improve women’s access to healthcare and satisfy their healthcare requirements? Methods include a case study in the Yuetan Area of Beijing, field investigation, mapping, and questionnaires. The survey was carried out in November 2021 and January 2022 and involved 462 women residents in the Yuetan Area. Results indicate: (i) that, despite the total number of facilities meeting the standards recommended by the WHO, the spatial distribution of healthcare facilities is imbalanced; (ii) women’s healthcare encompasses both physical and psychological health. Optimizing accessibility to healthcare facilities can positively impact women’s health and well-being. Conclusions include insights regarding the relationship between access to healthcare facilities women’s healthcare requirements, as well as proposing strategies for improved healthcare facilities with a focus on an equitable and sympathetic society.

## 1. Introduction

The concept of a Healthy City has been promoted by plenty of organizations and scholars since the 1980s. In health promotion, health is not considered an abstract condition, but as the ability of an individual to achieve her or his potential and to respond positively to the challenges of daily life [1]. The World Health Organization (WHO) describes a Healthy City as one that continually creates and improves its physical and social environments and expands the community resources that enable people to mutually support each other in performing all the functions of life and developing to their maximum potential [2,3]. The Healthy Cities Project, one of WHO’s major vehicles for achieving the strategy for health for all, attempts to strike a balance between placing health high on the strategic political agenda of cities and carrying out applied technical and operational measures [4]. This includes developing and implementing specific plans to improve health in the city and taking the structural, organizational and financial steps to make this possible [4,5]. 

Urban service facilities are a fundamental element of the convenience and efficiency of a healthy city [6]. As major components of urban service facilities, healthcare facilities have an essential impact on residents’ health and self-efficacy [7]. Health is among the most important services provided by governments of both developed and developing nations in the world, as the productivity of any one individual is dependent on their state of health [8]. According to Ogundare’s research, health has been likened to food in its importance to human existence. Ogundare further opines that the concern and attention that any government pays to health can determine the well-being of the people [9]. Healthcare facilities include hospitals, healthcare centers, clinics, and institutions. The accessibility of these facilities is a major indicator of development, and the spatial pattern of distribution of healthcare facilities can gauge the level of efficiency—or otherwise—of the provision of these facilities within any region [10].

Two significant factors influencing access to healthcare are healthcare facility supply and population demand [11]. The supply of healthcare facilities can be measured by both their density and spatial distribution in specific regions or areas. As the WHO indicates, healthcare facility density is primarily an index of access to outpatient services [12]. The recommended standard is 5 hospital beds per 1000 members of the population [13]. This ratio varies between countries and regions. For example, the number of acute hospital beds per 1000 people fell in the United States of America from 3.0 in 2000 to 2.5 in 2017 [14]. Over the same period, the comparable country average declined from 5.4 to 4.2 beds per 1000 people [14]. Spatial distribution is also a significant measure of healthcare facility supply. Furthermore, the healthcare sector rests not solely on service quality but also on the adequacy of health facilities [15]. Balanced spatial distribution of healthcare facilities enables accessibility, especially for the weak, sick, and disabled. Healthcare facility location has attracted considerable attention from the operations research community for close to four decades as one of the most important strategic issues in healthcare systems, disaster management, and humanitarian logistics [16]. Empirical studies in both developed and developing countries have linked inadequate access to healthcare facilities with increasing avoidable and preventable deaths [10].

Population demands for healthcare facilities have increased with the growth of cities [17,18,19]. Enhancing healthcare accessibility can contribute to addressing and accommodating the challenges facing burgeoning populations. Numerous recent studies conclude that the burden of harm resulting from the collective impact of all healthcare quality problems is staggering [20,21,22]. Governments may have huge budgets for fulfilling healthcare requirements; however, the implementation effects are not always as successful as expected. One reason is the long-standing neglect of appreciating the differences between the healthcare requirements of men and women. Women have distinct physical and psychological features [23]. Kiesel argues that the medical concepts of most diseases are based on understandings of male physiology, and women have altogether different symptoms than men [24]. The differences become especially distinct as people age. Based on Long’s research, six in ten (61%) women aged between 50 and 64 require regular monitoring, medical care, or medication [25]. Elderly women generally have as little as 37–68% of the muscle strength of males [26]. In later life, men can gradually lose an inch in height; women may lose about two inches [27]. Loss of height results in a lower center of gravity, making it difficult for elderly women to control their physical balance [23]. Long-term care issues affect women more often because they live longer; have higher rates of disability and chronic health problems; and lower incomes than men on average—putting them at greater need of state and community resources, such as medical aid [28]. Human beings, men, women, and gender diverse alike, are the center of concern for healthcare accessibility and all are entitled to a healthy and productive life [29].

According to the National Library of Medicine, US, women’s health refers to medicine focused on the treatment and diagnosis of diseases and conditions that affect a woman’s physical and emotional well-being [30]. Generally, women’s healthcare encompasses a wide range of areas such as gynecology, sexually transmitted infections (STIs), female-specific cancers, mammography, pregnancy and childbirth, sexual health, etc. [30]. Beyond biological differences, it is culture with its social structures including the gendered division of roles, societal functions, and social status that is a more influential factor in determining gender differences [31]. Women may experience more negative social psychology during healthcare than their male peers [23]. They often suffer more psychological pressure, a sense of inferiority, and alienation than men [32]. Safran argues that female patients may experience serious social bias [33]. Specifically considering women’s access to healthcare services and facilities thus makes a significant contribution to enhancing their quality of life.

**Figure 1:**
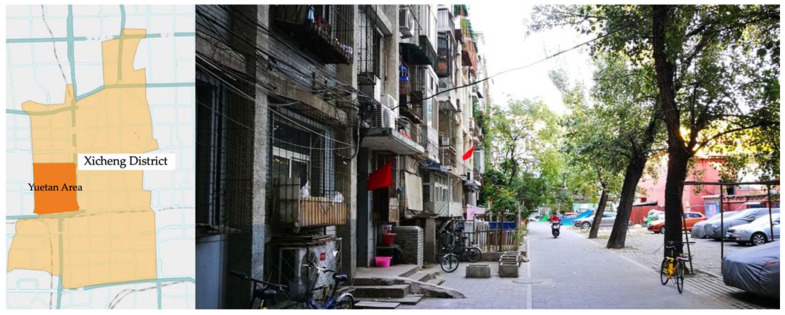
Location and neighborhood scenario of Yuetan Area.

Beijing is the site of the case study. The capital of China is home to more than 21 million residents and 16 urban, suburban, and rural districts [34]. Based on the Beijing Municipal Bureau of Statistics, the female population of Beijing has risen to 10.7 million [34]. In such a high-density, heavily populated city, the health and healthcare services provided to the female population relate directly to the development and sustainability of the city. The Yuetan Area of Beijing is the case study site of this research. Located in the Xicheng District, Yuetan Area is 6.5 km from the city center. According to the Beijing Municipal Bureau of Statistics, more than 150,000 residents live in Yuetan Area in total, of which about 70,100 residents are female [34]. Figure 1 presents the location of Yuetan Area in the Xicheng District and its street scenario. Yuetan Area is one of the oldest areas in Beijing. It has had a dense population and various commercial activities since the Ming and Qing Dynasties. In the old days of the Qing Dynasty, Yuetan Area was the place where emperors worshiped the moon. Contemporary buildings in this area began being constructed in the 1950s. Since then, roads and streets have been widened to accommodate a growing population and increased urban construction. Service facilities in the Yuetan Area are relatively diverse, including restaurants, retail, entertainment, education, travel agencies, and more. Five large-scale parking lots are clustered on the northeast side. There are nine bus stops and seven metro stations in Yuetan Area. A developed public transportation network facilitate people’s daily lives. Both residents and visitors could access Yuetan Park and the heritage Yuetan Temple conveniently by walking or in vehicles. 

This research focuses on women’s healthcare requirements and their access to healthcare facilities in urban areas. It has two major goals: (i) an analysis of healthcare facility density, spatial distribution and services in Yuetan Area, Beijing; (ii) an understanding of women’s healthcare requirements and the changes in healthcare facilities they need. As a result of this analysis, strategies are proposed for creating more equitable healthcare facilities and services. The research question asks: What strategies will improve women’s access to healthcare and satisfy their healthcare requirements? The extent to which healthcare facilities contribute to sustainable urban societies is also analyzed, assessing the real needs and expectations of women for their well-being across different life stages. 

## 2. Materials and Methods

Close to five hundred female residents from Yuetan Area were invited to participate in this research. The survey sample accounts for 0.71% of the total female population of various ages and took place over 2 months between 1 November 2021 and 1 January 2022. This paper limits the research objects to the healthcare facilities and women’s healthcare requirements.

**Figure 2:**
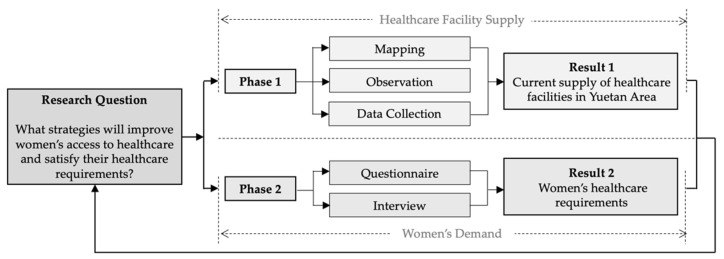
Methodological framework.

The methodological framework consists of two phases (Figure 2): 

Phase one comprises mapping, observation, and data collection in Yuetan Area. By recording spatial distributions, mapping locates existing healthcare facilities. Observation records the healthcare services that these facilities can provide and notes any services specific to female patients. Data collection enables an understanding of the density and scale of healthcare facilities, as well as the number of households and extent of the population serviced. Results are indicative of the current supply of healthcare facilities in Yuetan Area. 

Phase two consists of questionnaires and interviews to explore the healthcare needs of women. Women’s medical habits, preferences and expectations for healthcare were asked and recorded. Results include a deep analysis of women’s healthcare requirements. Finally, strategies for improving healthcare accessibility for women are proposed to address the research question.

### 2.1. Mapping, Observation and Data Collection

Mapping, observation, and data collection are employed to gain an understanding of the current supply of healthcare facilities in Yuetan Area. Mapping is the process of locating each healthcare facility on one site map. Noting all the healthcare facilities on one map of Yuetan Area enables analysis of the locations, scale, and spatial distribution of the existing supply. The observation method is described as a method to observe and describe the behavior of a subject [35]. In this research, observation describes healthcare services specifically provided to female patients or customers. This method collects information by watching healthcare activities and recording them for analysis in a later phase. Observation of the healthcare facilities in Yuetan Area included recording medical staff compositions, gynecological B-ultrasound services, and cervical cancer screening services. Data collection is the third step of Phase one. According to Kabir’s research, data collection is the process of gathering and measuring information on variables of interest, in an established systematic fashion that enables one to answer stated research questions [36]. Collected data are analyzed by counting the numbers of healthcare facilities, calculating the population and community areas, as well as classifying the existing healthcare facilities. Data summarizing population, households, community areas, and service areas enable analysis of the density, service coverage, and service capability of healthcare facilities were collected. 

### 2.2. Questionnaire and Interview

Questionnaires and interviews were the survey techniques used to explore women’s healthcare requirements. Questionnaires consist of questions or other types of prompts that aim to collect information from a respondent [37]. During the field study, questionnaires were targeted to gain an understanding of the participants’ satisfaction with current healthcare facilities. There were 462 valid questionnaires returned from the Yuetan Area, accounting for 92.4% of total questionnaires distributed. Of the 70,100 female residents of Yuetan Area [34], approximately 0.65% female residents validly participated in the survey. The questionnaire addressed a range of themes. It comprised three sections and included closed-and open-ended survey questions. 

Section one collected basic information, including age, health status, community, work, etc. This information forms a picture of each participant’s current situation.

Section two addresses healthcare needs. It includes questions to ascertain what kinds of disease research participants may have experienced—or be currently experiencing; how often they visit a healthcare facility; the nature of their preferred healthcare facility; common healthcare needs, etc. This information explores the characteristics of women’s healthcare types and needs.

Section three investigates women’s satisfaction with, and expectations of, healthcare facilities. It includes Likert Scale questions related to satisfaction with current healthcare facilities and psychological motivations behind visiting a healthcare facility. This section analyzes what kinds of healthcare facilities are accessible to women. 

Interviewing is a qualitative survey technique that involves asking open-ended questions to facilitate question–answer conversation with the women of Yuetan Area. Interviews were unstructured, with a thematic approach rather than a rigid question structure [38]. The purpose of interviews was to record women’s attitudes to healthcare and healthcare facilities. Respondents answered the questionnaire and interview questions anonymously. Data security and privacy were protected and used exclusively for the purpose of this academic research. Ethical approval was officially given.

## 3. Results and Discussion

### 3.1. Current Healthcare Facility Supply in Yuetan Area

**Figure 3:**
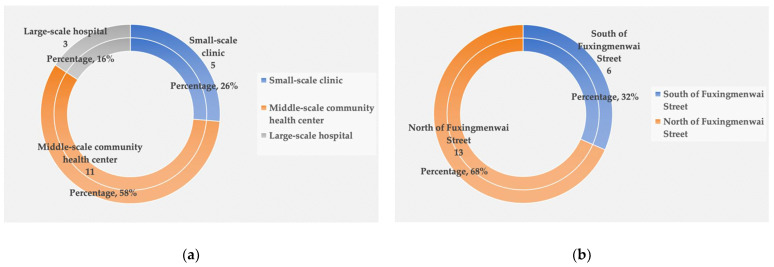
Density of healthcare facilities: (**a**) number of each type of healthcare facility; (**b**) spatial distribution of healthcare facilities.

**Figure 4:**
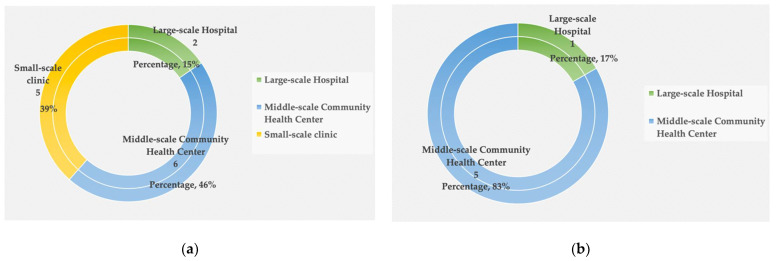
Number of healthcare facilities: (**a**) number of each type of healthcare facility on the north side Fuxingmenwai Street; (**b**) amount of each type of healthcare facility southside of Fuxingmenwai Street.

**Figure 5:**
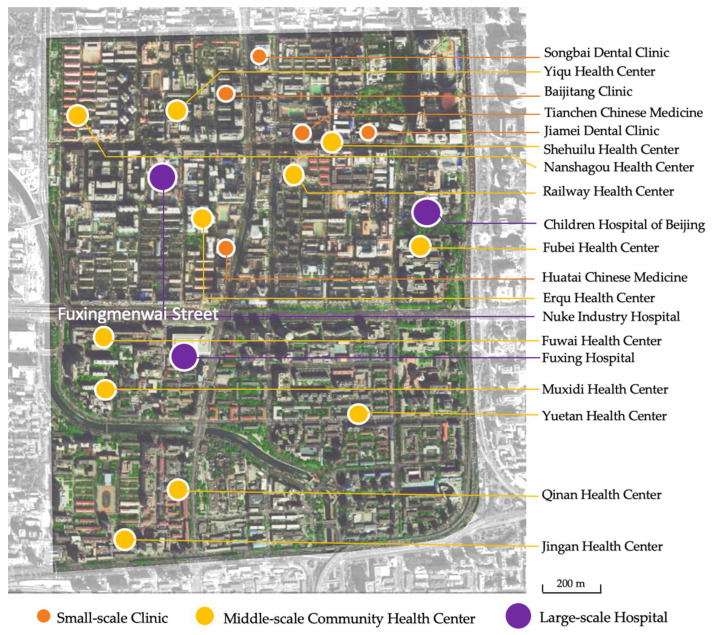
Healthcare facilities in Yuetan Area.

Findings indicate that healthcare facilities consist mainly of three types. These are: small-scale clinics, middle-scale community health centers, and large-scale hospitals. There are 19 healthcare facilities of different scales serving more than 150,000 residents in Yuetan Area. As Figure 3a demonstrates, the 11 community health centers make up the highest percentage (58%) of facilities. Clinics are second, at 26% and hospitals 16%. This suggests that community health centers are the primary choice available to patients. Location-wise, Fuxingmenwai Street forms a natural north–south boundary as it is located at the approximate geographical centerline of Yuetan Area. As Figure 3b indicates, most healthcare facilities are situated at the north of Fuxingmenwai Street, accounting for 68% of the total number of facilities. Other facilities are distributed along the south of Fuxingmenwai Street. Figure 4a shows that residents living in the north of Fuxingmenwai Street can receive healthcare services from two hospitals, six community health centers, or from five small-scale clinics. However, healthcare facilities in the south of Fuxingmenwai Street are comparatively fewer—one hospital and five community health centers, as seen in Figure 4b. Populations on both sides are comparable on; the northside is home to six communities and the southside five. The results of mapping healthcare facilities in Figure 5 indicate an imbalanced trend of more healthcare facilities for northside residents and fewer for those on the south. There is also a lack of small-scale healthcare facilities on the southside. 

Community health centers make up the highest percentage of healthcare facilities. These are non-profit practices organized by the local government. Table 1 presents the areas and services provided by each community health center. Information of Table 1 was collected from Yuetan Community Committee and the Beijing Municipal Bureau of Statistics. Yuetan Community Committee provides the areas of each health center during the interview of data collection. The Beijing Municipal Bureau of Statistics provides the numbers of households and population in their formal report for public access. These health centers are 6135 m^2^ in total area, and service approximately 48,405 households. Small-scale clinics can supplement other healthcare service providers and are especially accessible to low-income families. The large-scale hospitals in the Yuetan Area include a specialized children’s hospital and two general hospitals. They are municipal-level facilities, open to the entire city rather than to specific communities.

In the Yuetan Area, hospital beds per 1000 members of the population is about twice that of the standard recommended by the WHO. However, women-specific healthcare services are insufficient. For example, only seven healthcare facilities can provide services specifically to women patients or customers, and five healthcare facilities can provide gynecological B-ultrasound examination services −26% of the total facilities. Three healthcare facilities (16%) have the capability to provide cervical cancer screening services. Although Chinese healthcare resources have developed significantly to cater for rising demand [39], the imbalanced distribution of healthcare resources has created inequality between genders.

### 3.2. Women’s Healthcare Requirements 

**Figure 6:**
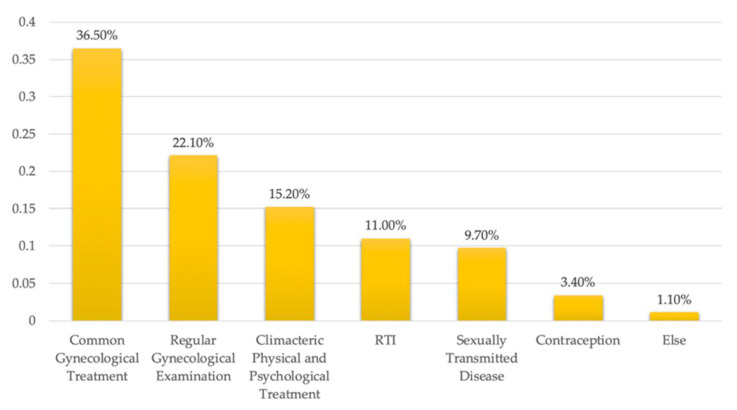
Different healthcare requirements of women in Yuetan Area.

The healthcare requirements of female residents in Yuetan Area include common gynecological treatment, regular gynecological examinations, climacteric physical and psychological treatments, reproductive tract infections (RTI), etc. Figure 6 indicates the percentage spread across women’s healthcare needs. Common gynecological treatment accounts for 36.50% of women’s healthcare requirements, ranking first in demand. Of the 462 questionnaire responses, 169 participants required healthcare for common gynecological complaints, such as cervicitis and ovarian cysts. A total of 102 participants, (22.10%), have regular gynecological examinations to ensure that their bodily functions are in a healthy state. According to the WHO, population aging and urbanization are two global trends that, together, are major forces shaping the twenty-first century [40]. As of 2020, the elderly population (older than 65) in Beijing had risen to 2.9 million [34]. With the aging population increasing, elderly women patients become an essential group for healthcare services. In Yuetan Area, 70 participants, 15.2% of the total, require climacteric physical and psychological treatment. Other needs, including those related to sexually transmitted disease, contraception, obstetric inspection, and sex psychology were also raised in the survey.

**Figure 7:**
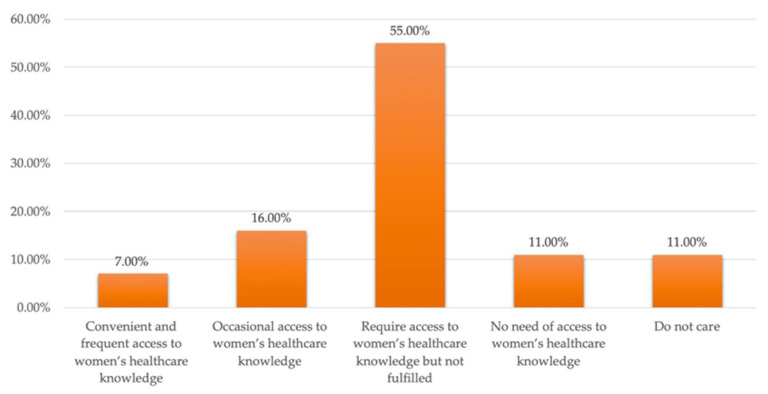
Women’s accessibility to healthcare knowledge.

Results indicate that participants are also in need of access to knowledge about women’s health. Just six healthcare facilities in Yuetan Area provide free consultations or flyers detailing healthcare knowledge—strongly suggesting that women’s needs in this arena are not being met. It was far fewer for 70,100 women in Yuetan Area [34]. As Figure 7 presents, about 254 participants, 55% of total participants, claimed that more women’s health knowledge was needed but is unavailable. Only 32 participants, 7% of the total sample, indicated that they have convenient and frequent access to women’s healthcare knowledge.

**Figure 8:**
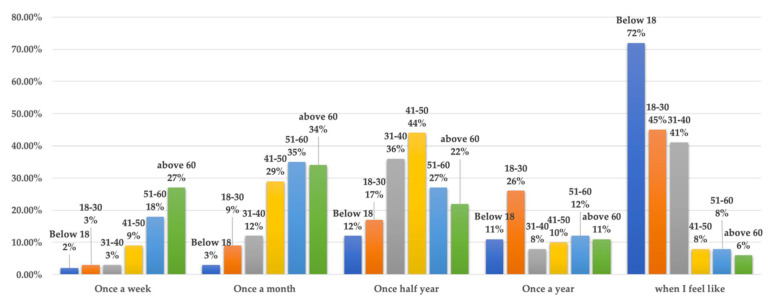
Frequency of receiving healthcare services of women across different ages.

Yuetan Area is typical of the aging population of Beijing. Approximately 14,020 elderly women (aged above 65) live in Yuetan Area [34]. Of these elderly women, 92 participated in this research and around 60% of those received medical treatment or healthcare services in the previous two weeks. Elderly women generally require more involved medical treatment. This is due not only to the gradual decline of physical functions, but also because elderly women are more likely to suffer psychological problems than their male peers [23]. Young women (18 years older younger) in comparison, were treated infrequently. About 72% of women younger than 18 years old choose to receive healthcare services when they feel they need them, rather than regularly. Young women tend to believe that their physical condition is good, and they rarely experience physical discomfort. When faced with a little discomfort, they prefer to buy medicines without seeing doctors. Figure 8 indicates the frequency of receiving healthcare services across different ages.

**Figure 9:**
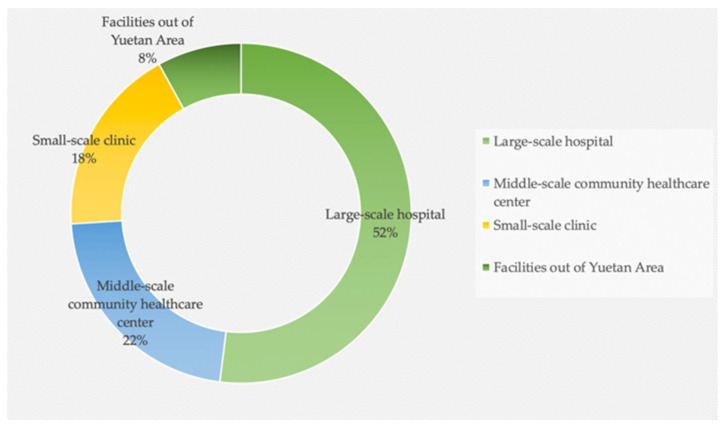
Healthcare service selections of women.

The choice of healthcare facilities by female patients or consumers is also an indicator of healthcare accessibility. There were 97 of the participants (21%) which would like to visit a healthcare facility when they experience mild discomfort, such as palpitations, dizziness, insomnia, etc. Most participants preferred to rest rather than see a doctor. Shyness about gynecological disorders exacerbates reluctance to see a doctor. This value increased to 314 (68%) when participants suffered serious disease such as extremity edema, abnormal leucorrhea, inflammation, and irregular menstruation. About 51 participants avoid visiting a healthcare facility due to shyness, psychological barriers, or for other reasons. As Figure 9 demonstrates, more than half of all participants preferred large-scale hospitals because they believe these have advanced medical equipment, superior doctors, and a richer clinical experience. An amount of 22% choose community healthcare centers for reasons of economy and convenience. A small number of participants preferred small-scale clinics for reasons such as privacy, and about 8% of participants preferred healthcare facilities outside of Yuetan Area, such as specialized facilities or other top standard hospitals. 

**Figure 10:**
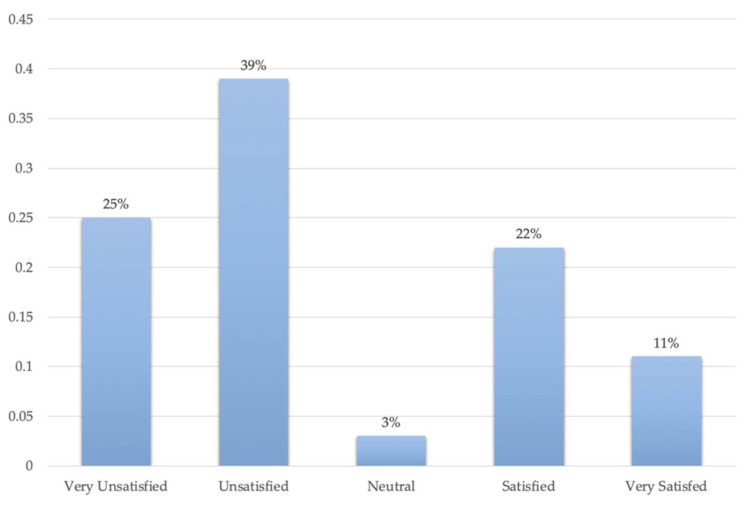
Women’s satisfaction with healthcare facilities.

This research used the Likert method to investigate women’s satisfaction with healthcare facilities. The results are polarizing. Most participants were unsatisfied and very unsatisfied with current healthcare facilities, while a considerable number of participants were satisfied or very satisfied. This finding confirms, to some extent, the imbalance of healthcare facility supply and delivery. Figure 10 presents the participants’ satisfaction with the healthcare facilities of Yuetan Area. Most women—up to 39%—chose the “unsatisfied” option. About 25% chose the “very unsatisfied” option. Participants who chose “unsatisfied” and “very unsatisfied” complained about different aspects of the healthcare facilities. First is the long distance from home or workplace to the healthcare facilities. They find healthcare facilities are too far away to access. As a result, many women would rather rest at home than visit a facility, especially when they feel too uncomfortable to move. Second is the lack of obstetrics and gynecology services. Some participants tend to visit close community health centers; however, most community health centers do not provide obstetrics and gynecology medical staff, or screening instruments. Third is inaccessible healthcare knowledge. Some participants believed that healthcare facilities have a responsibility to publicize and disseminate information about women’s healthcare, as well as care for women’s health. However, most healthcare facilities of Yuetan Area do not offer this service. Conversely, some participants chose the “satisfied” (22%) or “very satisfied” (11%) options. Part of them found the number of healthcare facilities in Yuetan Area relatively accessible compared to other areas or cities. Some communities have high-density clinics and community health centers for female patients. 

## 4. Strategies to Improve Healthcare Facilities’ Accessibility

Based on the survey results and analysis above, this research proposes three strategies to improve the accessibility of healthcare facilities in Yuetan Area to better meet the healthcare requirements of women. 

First is optimization of the spatial distribution of healthcare facilities. Although the total number of healthcare facilities in Yuetan Area can meet the needs of the current population, the spatial distribution of these facilities has not been fully considered. Some residents can access healthcare facilities conveniently; others are excluded from their nearest healthcare practice. As the spatial distribution of urban service facilities is largely constrained by the road network [39], the local government has a responsibility to balance urban service facilities, including healthcare facilities, from the early stages of urban planning and design to ensure that most residents can access healthcare facilities equally. The Office of the Victorian Government Architect believes that of all public facilities, hospitals and medical centers should be the ones that are built with the greatest care and imagination [41]. For female patients, juggling work and family fills their daily routines so much that they can only seek healthcare from the nearest facilities or take a short break when discomfort occurs. A balanced spatial distribution of healthcare facilities helps to improve equality of healthcare facility supply. 

Secondly, providing healthcare services specifically for female patients and customers. The survey results indicate that healthcare services for women are relatively lacking. Current services cannot meet the requirements of women’s healthcare. Several barriers can make it harder for women to access critical healthcare services or build optimal working relationships with their providers [42]. Harvard University organized the Women and Health Initiative to collaborate with communities and stakeholders on women and health [43]. Healthcare facilities, especially community health centers, can improve their service capability by employing measures such as obtaining accessible healthcare equipment and screening machines, adding obstetric and gynecological care to their outpatient services, and providing alternative communication methods when requested to avoid shyness preventing access to care. 

Finally, closely coordinating healthcare facilities and women, survey participants are eager to receive healthcare and basic knowledge of women’s health. Across communities, the range of healthcare facilities needed and the ability of individuals to access healthcare services varies widely [44]. Community committees can take responsibility for maintaining relationships between healthcare facilities and women residents. For example, they may hold formal or informal community meetings with both women residents and healthcare facilities. Such meetings can the voice of women residents to be heard, enable healthcare facilities to better understand women’s healthcare needs and improve the accessibility of healthcare facilities. Accessible healthcare facilities are an essential requirement, benefitting from extensive participation from society [45].

## 5. Conclusions

This research highlights the need for healthcare facilities to specifically address women’s special healthcare requirements, with a view towards equality and sympathy. Research undertaken in the Yuetan Area of Beijing indicates that the existing healthcare facility supply has challenges in terms of density, spatial distribution and services. Imbalanced healthcare planning and a lack of specific healthcare for women diminish the accessibility of healthcare facilities. In addition, a questionnaire survey and qualitative and quantitative data analysis were conducted on more than four hundred female residents. Women’s healthcare requirements were ascertained, and found to include, among others, gynecological services and dissemination of healthcare knowledge. Women’s satisfaction with current healthcare facilities has also been explored. Results show that a considerable number of women are dissatisfied with the existing healthcare facilities. The need for healthcare facilities to improve their services for women is urgent. According to the above analysis of healthcare facility supply and women’s needs, three strategies for improving the accessibility of healthcare facilities are proposed, including (i) optimizing the spatial distribution of healthcare facilities; (ii) providing healthcare services specifically for female patients and customers; and (iii) closely coordinating healthcare facilities and female residents. These strategies support the healthcare facility system enhancement and well-being of women at every life stage. Promoting women’s physical and mental health from the perspective of urban governance, urban design and public facility reconfiguration should be further studied in future.

## Figures and Tables

**Table 1:** Areas of health centers and service targets of Yuetan Area.

No.	Name	Area (m^2^)	Service Targets		
			Community Area (km^2^)	Households	Population
1	Yuetan Health Center	2380	1.19	19,630	33,528
2	Yiqu Health Center	840	0.71	5860	21,474
3	Erqu Health Center	259	0.49	4056	13,562
4	Muxidi Health Center	689	0.42	3584	15,290
5	Fuwai Health Center	120			
6	Shehuilu Health Center	183	0.28	3983	12,441
7	Nanshagou Health Center	120	0.1	474	1292
8	Fubei Health Center	360	0.61	4611	17,029
9	Jingan Health Center	224	0.16	2584	7196
10	Qinan Health Center	160			
11	Railway Health Center	800	0.15	3623	11,626
	total	6135	4.11	48,405	121,812

Source: Yuetan Community Committee and the Beijing Municipal Bureau of Statistics [34].
